# Excess glucose inhibits the cotyledon greening of etiolated seedlings

**DOI:** 10.1080/15592324.2023.2191465

**Published:** 2024-11-14

**Authors:** Zi-Meng Yao, Hu-Hui Chen

**Affiliations:** a School of Horticulture, Nanjing Agricultural University, Nanjing, Jiangsu, People’s Republic of China; b School of Life Science, Nanjing Agricultural University, Nanjing, Jiangsu, People’s Republic of China

**Keywords:** SUCROSE TRANSPORTER 2 (SUC2), De-etiolation, PHYTOCHROME A (PHYA), ETHYLENE-INSENSITIVE3 (EIN3), Glucose signaling

## Abstract

The capability of the transition from skotomorphogenesis-to-photomorphogenesis (de-etiolation) is requisite for seedling survival and development. However, how carbohydrate in germinating seeds controls seedling de-etiolation remains unclear. Mu et al. (2022) investigated the regulatory roles of soluble sugars (such as, glucose or sucrose) on de-etiolation during the transition from skotomorphogenesis-to-photomorphogenesis. The authors revealed that in the dark, sucrose/glucose in germinating seeds induces ethylene production/signaling. Ethylene signaling promotes the stability of EIN3 (ETHYLENE-INSENSITIVE3), a key component in the ethylene signaling pathway. In turn, EIN3 directly binds to the promoter of *SUC2* (*Sucrose Transporter 2*), encoding a major sucrose transporter, to repress *SUC2* transcription. The resulting phloem loading of sucrose is blocked, and thereby the accumulation of sucrose is elevated in etiolated seedling cotyledons. When exposed to light irradiation, accumulated sucrose/glucose inducing ethylene elevates the stability of EIN3, repressing *phyA* (encoding the photoreceptor of a far-red light/the inhibitor of a cotyledon greening) expression to promote de-etiolation. In this study, we mainly discuss the findings (low sugars promote de-etiolation) of Mu et al. (2021) and further find that excess sugars inhibit de-etiolation.

The cotyledon greening of etiolated seedlings (de-etiolation) is requisite for plant survival and development. In the life cycle of plants, early seedling establishment, seed germination and de-etiolation all are depending on storage substances primary carbohydrates, being transported in the form of soluble sugars (sucrose, glucose and fructose) from storage seed organs to a lot of organs like hypocotyls, radicles and cotyledons^1^,^2^. Mu et al.^3^ first revealed molecular mechanism of how carbohydrate signaling controls de-etiolation.

Mu et al.^3^ revealed that an ACS7—EIN3—SUC2 module under darkness represses carbohydrate partitioning through inhibiting sucrose retrieval along the transport phloem in etiolated seedling cotyledons, and thus accumulates carbohydrates within these cotyledons to induce constant ethylene production to preserve high EIN3 activity. When these etiolated seedling cotyledons are exposed to light, high EIN3 activity directly blocks the function of *phyA*, encoding the photoreceptor of a far-red light/the inhibitor of cotyledon greening to promote de-etiolation. Authors propose that low sugar availability facilitates de-etiolation through sugars stimulated-ethylene inhibiting both the photoreceptor of a far-red light and the partitioning of carbohydrate. This study thus elucidates how carbohydrate induced-ethylene directly and negatively controls the photoreceptor of a far-red light and the partitioning of carbohydrate to promote de-etiolation through both EIN3—phyA and ACS7—EIN3—SUC2 modules.

Whereas sugar accumulation is requisite for *ACS7* [1-amino-cyclopropane-1-carboxylate synthase (*ACS7*, encoding an important ethylene biosynthesis enzyme)] expression, ethylene production and potentially de-etiolation under subterranean darkness, the issue of if glucose or other sugars regulate this process and if these sugars modulate this physiological process through metabolism and/or signaling remains to be determined. Mu et al.^3^ found that both exogenous glucose and sucrose, but not mannose, promoted ethylene production/*ACS7* expression and de-etiolation. *vinv2* is a sugar osmotic stress mutant^4^. While glucose level in *vinv2* mutant was reduced, the values of its ethylene production and de-etiolation were not significantly different relative to those of wild-type seedlings, indicating that the decline of osmotic stress activities which causes the decrease of glucose levels cannot interrupt glucose signaling induced-de-etiolation. Furthermore, the sugar signaling/metabolism mutant *cinv1/2*^2,5^ had reduced or increased^1,2^ endogenous glucose contents under different conditions, thereby decreasing ethylene production and in turn leading to failing de-etiolation, further implying glucose signaling, rather than metabolism, promotes de-etiolation. This is because both ethylene production and de-etiolation are not dependent on glucose level in *cinv1/cinv2* mutant seedlings. Further, the reduction in ethylene production and the failure of de-etiolation can be restored via glucose, but not sucrose and mannose, strongly implying that sugars promote both *ACS7* expression/ethylene production and de-etiolation through initiating glucose signaling. Therefore, following seed germination, its soluble sugars positively regulate both *ACS7* expression/ethylene production and de-etiolation through initiating glucose signaling.

Under subterranean darkness, de-etiolation is promoted under conditions of low soluble sugar availability. Therefore, soluble sugars represent a novel class of regulators that function in conjunction with ethylene signaling, sucrose transporter and the photoreceptor of a far-red light following seed germination to optimize de-etiolation of *Arabidopsis* seedlings as they emerge from subterranean darkness and exposed to light.

However, it is unclear whether excess glucose blocks de-etiolation when sugar accumulation reaches a certain level. In this study, we focus on this. When sucrose/glucose accumulation reaches a certain level via the ACS7–EIN3–SUC2 module, excess glucose can promote EIN3 instability in cotyledons^6^, which in turn might block de-etiolation via the EIN3–PHYA module. Indeed, 7-d-old wild-type (Col-0) etiolated seedlings grown on solid MS medium with either 2.0% or 4.0% Sucrose/Glucose/Mannose or without were transferred to white light for 12 h. The greening rate of these etiolated seedlings was obviously elevated under 2.0% Sucrose/Glucose, but not 2.0% Mannose, whereas the greening rate was significantly reduced 4.0% Sucrose/Glucose (Figures 1a,b).

**Figure 1.**
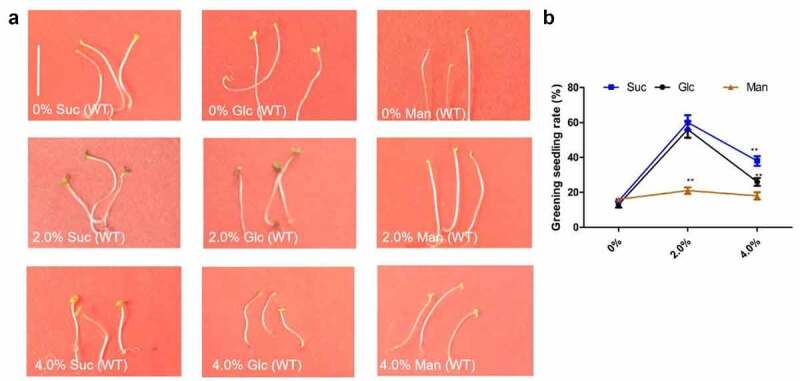
Cotyledon greening of etiolated seedling was promoted under low glucose/sucrose, but was suppressed under excess glucose/sucrose.

(a) Seven-d-old wild-type (Col-0) etiolated seedlings followed by 12 h of normal white light irradiation on solid MS medium with 0%, 2.0% and 4.0% sucrose (Suc), glucose (Glc) and mannose (Man) or without. Bars = 1 cm. Material with similar phenotype was observed at least 50 independent wild type.

(b) Bar graph illustrating the greening rate in (a).

Error bars represent SD (n = 50). Student’s *t* test (**P < 0.01).

To test EIN3 activity mediated via excess glucose, we used a transgenic line containing the *GUS* reporter gene downstream of the five tandem repeats of EIN3 binding site (EBS) followed by a minimal 35S promoter (*5×EBS:GUS*). The system is used for detecting *EIN3* activity^7^. In the representative *5×EBS:GUS* transgenic plants treated with 4% glucose for either 3 or 6 h, the intensity of GUS staining decreased in the presence of light (Figure 2a). Further, as was shown in Figure 2b, the quantification of GUS activity was tested. Our findings of the GUS intensity were reduced with increasing glucose. By contrast, no decline was detected by following treatment with 4% mannose (Figures 2a,b). This result implied that excess glucose declined *EIN3* activity, and this phenomenon is independent of any potential effects on osmotic stress. This finding also indicated that excess glucose represents a stress, which makes against de-etiolation.

**Figure 2.**
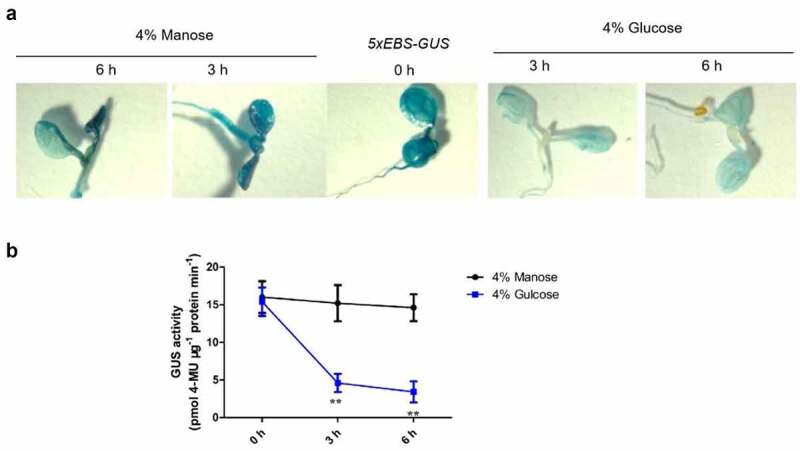
EIN3 activities were suppressed by excess glucose but not mannose.

(a) Etiolated seedling cotyledons of *5×EBS* (*EIN3 binding site*)-*GUS* were grown on solid MS medium with 2% sucrose for 7 d and then treated with 4% glucose or 4% mannose for 3 or 6 h. Subsequently, GUS staining was performed.

(b) Expression of the *5×EBS-GUS* reporter gene in etiolated seedling cotyledons of seedlings grown on MS medium with 2% sucrose for 7 d under light was assayed quantitatively at the indicated time point via determining GUS activity. This activity was measured by picomoles of 4-methyl umbelliferone (4-MU) per mg protein per min. Error bars represent SD (n = 3). Student’s *t* test (**P < 0.01).

Together, our findings as well as the findings of Mu et al. (2021) indicate that de-etiolation is promoted under conditions of low soluble sugar availability, whereas de-etiolation is blocked under excess sucrose/glucose.

## Methods and materials

Seeds of *5×EBS:GUS* were kindly provided through Prof Zi-qiang Zhu (Nanjing normal university, Nanjing, China). Growth conditions were maintained as described by Mu et al^3^. GUS assay was performed as described by Meng et al^8^. Histochemical assay of GUS activity was performed, as has been described by Li et al^7^. The cotyledon greening of etiolated seedlings was analyzed and performed, as was described by Mu et al (2021).
